# Association of muscle mass and fat mass with insulin resistance and the prevalence of metabolic syndrome in Korean adults: a cross-sectional study

**DOI:** 10.1038/s41598-018-21168-5

**Published:** 2018-02-09

**Authors:** Kyuwoong Kim, Sang Min Park

**Affiliations:** 1 Department of Biomedical Sciences, Seoul National University Graduate School, Biomedical Science Building 117, Daehakro 101, Jongro-gu, Seoul, 110-744 Republic of Korea; 2 Department of Family Medicine, College of Medicine, Seoul National University, Daehak-ro 101, Jongno-gu, Seoul, 110-744 Republic of Korea

## Abstract

Relationship of muscle mass and fat mass with insulin resistance and metabolic syndrome remains uncertain, especially among Asian population. We performed a cross-sectional study with 14,807 adult participants aged between 18 and 65 in the fourth and fifth Korea National Health and Nutrition Examination Survey with Dual Energy X-ray Absorptiometry (DEXA) data to investigate whether muscle mass and fat mass are associated with insulin resistance and metabolic syndrome. DEXA records were used to categorize the participants into four categories (low muscle/low fat, low muscle/high fat, high muscle/ low fat, and high muscle/high fat). Least square means and incidence rate ratios (IRR) were used to assess the associations of muscle mass and fat mass with insulin resistance and metabolic syndrome. After adjustment for potential confounders, high muscle/low fat was associated with significantly lower insulin resistance (*P* < *0.001*) compared to low muscle/low fat. Low muscle/high fat (IRR: 1.90; 95% confidence interval [CI]:1.44–2.50, *P* < *0.001*) and high muscle/high fat (IRR: 2.30; 95% CI:1.76–3.00, *P* < *0.001*) were significantly associated with the prevalence of metabolic syndrome. Our study suggests that protective association of muscle mass with metabolic syndrome is attenuated by high fat mass in Korean adults.

## Introduction

Although body mass index (BMI) has been widely used as a significant predictor for diabetes mellitus, hypertension, and dyslipidemia^1,2^, the relationships of muscle mass and fat mass to insulin resistance and metabolic syndrome is not well established. Some epidemiological studies have shown that BMI level alone may lack predictive value for type 2 diabetes due to differences in muscle mass and fat mass (particularly abnormal adiposity) for the same BMI unit^3,4^.

In the United States, the National Health and Nutrition Examination Survey (NHANES) conducted from 2003 to 2006 showed that the BMI was positively associated with the prevalence of metabolic syndrome based on the National Cholesterol Education Program’s Adult Treatment Panel III (NCEP/ATP III) criteria^5^. In that study, both overweight and obese women were more likely to meet the criteria for metabolic syndrome compared to those in the normal weight range. However, Palaniappan *et al*. showed that higher BMI was not associated with prevalence of metabolic syndrome among Asian patients based on electronic health records^6^. While ethnicity may explain these inconsistent reports along with other factors, it is not yet entirely clear whether the different results are due to ethnic differences in BMI and body fat percentage relationship^7,8^.

A cross-sectional study showed that muscle and body fat compartments such as subcutaneous adipose tissue (SAT) and visceral adipose tissue (VAT) may be a more clinically important predictor of metabolic syndrome than BMI^9^. Furthermore, recent studies suggest that evaluating body muscle and fat content instead of BMI might fill the knowledge gap between high BMI and unexpectedly improved health outcomes^10,11^. Therefore, it is important to clarify the association of muscle mass and fat mass with insulin resistance and the prevalence of metabolic syndrome.

We used a nationally representative study sample to investigate the relationships of muscle mass and fat mass to insulin resistance and metabolic syndrome among Korean adults.

## Results

Table 1 shows the characteristics of the participants in our study by muscle mass and fat mass. Among 14,807 participants, 4,591 participants were classified as low muscle/low fat group, 2,311 as low muscle/high fat group, 2,956 as high muscle/low fat group, and 4,949 as high muscle/high fat group, respectively. Except for the low muscle/low fat category, the percentage of men and women were similar across the different categories of body composition types. The mean (standard deviation) of the BMI was the highest among high muscle/high fat participants 26.8 (2.59) kg/m^2^, and the lowest among low muscle/low fat participants 20.4 (2.16) kg/m^2^. The differences in age, sex, smoking status, alcohol consumption, physical activity, BMI, and presence of comorbidity were statistically significant (p < 0.05 for all comparisons) across the four muscle mass and fat mass groups.

**Table 1:** Sociodemographic, health-related, and clinical characteristics of the participants by muscle mass and fat mass among the participants of the KNHANES IV-V from 2008 to 2011.

Characteristics	Low Muscle/ Low Fat (*N* = *4,591*)	Low Muscle/ High Fat (*N* = *2,311*)	High Muscle/ Low Fat (*N* = *2,956*)	High Muscle/ High Fat (*N* = *4,949*)	P-value
Sex, n(%)					<0.001
Male	1,655 (36.1)	1,015 (43.9)	1,438 (48.7)	2,255 (45.56)	
Female	2,936 (63.9)	1,296 (56.1)	1,518 (51.3)	2,694 (54.44)	
Age	39.4 (12.6)	47.9 (11.8)	40.4 (12.0)	45.9 (11.8)	<0.001
Education Level, n(%)					<0.001
≤Elementary	462 (10.1)	461 (20.1)	310 (10.5)	1,007 (20.4)	
Middle/High School	2,370 (52.0)	1,144 (49.8)	1,625 (55.2)	2,495 (50.6)	
≥University/College	1,730 (37.9)	692 (30.1)	1,010 (34.3)	1,427 (29.0)	
Employment Status, n(%)					<0.001
Employed	2,794 (61.5)	1,459 (63.6)	2,048 (69.9)	3,317 (67.6)	
Unemployed^†^	1,751 (38.5)	834 (36.4)	881 (30.1)	1,588 (32.4)	
Residential Area, n(%)					0.002
Capital	1,260 (27.5)	599 (25.9)	743 (25.1)	1,202 (24.3)	
Metropolitan	964 (21.0)	455 (19.6)	662 (22.4)	1,022 (20.7)	
Town/City	2,367 (51.5)	1,257 (54.4)	1,551 (52.5)	2,725 (56.0)	
Household Income, n(%)					<0.001
Lowest Third	530 (11.7)	284 (12.4)	274 (9.4)	629 (12.8)	
Middle Third	2,575 (56.8)	1,269 (55.5)	1,679 (57.5)	2,834 (57.8)	
Highest Third	1,427 (31.5)	734 (32.1)	965 (33.1)	1,438 (29.4)	
Smoking Status, n(%)					<0.001
Non-Smoker	2,917 (63.9)	1,332 (57.9)	1,690 (57.4)	2,846 (57.7)	
Former Smoker	994 (21.8)	475 (20.7)	716 (24.3)	1,107 (22.4)	
Current-Smoker	657 (14.3)	493 (21.4)	541 (18.3)	983 (19.9)	
Alcohol Consumption, n(%)					<0.001
Non Drinker	1,936 (43.4)	1,007 (44.2)	1,068 (36.7)	2,047 (41.8)	
Drinker	2,524 (56.6)	1,274 (55.8)	1,843 (63.3)	2,848 (58.2)	
Sleep Duration (h/day)	7.05 (1.31)	6.87 (1.30)	6.85 (1.25)	6.78 (1.28)	<0.001
Physical Activity (MET-min)*					<0.001
Low (≤600)	1,601 (35.2)	756 (33.0)	713 (24.3)	1,377 (28.0)	
Moderate (601~2,999)	1,873 (41.1)	980 (42.8)	1,162 (39.6)	2,067 (42.0)	
High (≥3,000)	1,080 (23.7)	554 (24.2)	1,062 (36.1)	1,477 (30.0)	
Body Mass Index (kg/m^2^),	20.4 (2.16)	23.7 (1.69)	22.9 (1.69)	26.8 (2.59)	<0.001
Trunk Fat Mass (kg),	6.18 (1.82)	11.01 (2.03)	6.67 (1.77)	12.1 (2.85)	<0.001
Appendicular Muscle Mass (kg),	15.7 (3.67)	16.3 (3.89)	20.0 (4.69)	20.1 (4.86)	<0.001
Nutritional Intake,					
Total Energy Intake (kcal/day)	1930 (806)	1866 (772)	2132 (930)	2008 (905)	<0.001
Vitamin C (mg/day)	106 (91)	106 (102)	120 (102)	113 (94.6)	
Niacin (mg/day)	16 (8.67)	15.9 (8.54)	18.1 (10.3)	17.1 (10.2)	
Sodium (g/day)	4.79 (3.00)	4.78 (2.96)	5.25 (3.19)	5.20 (3.54)	
Calcium (g/day)	0.49 (0.31)	0.49 (0.31)	0.56 (0.35)	0.52 (0.34)	
Fiber (g/day)	7.19 (5.17)	7.62 (5.51)	7.99 (5.37)	8.05 (5.97)	
Cardiometabolic Status					
Fasting Serum Glucose	91.6 (18.9)	98.4 (21.9)	92.5 (16.3)	100 (23.1)	<0.001
Waist Circumference	72.0 (7.29)	82.3 (6.66)	77.6 (6.50)	88.7 (7.89)	
Systolic Blood Pressure	110 (15.0)	119 (16.4)	114 (15.3)	121 (16.1)	
Diastolic Blood Pressure	73.1 (10.2)	78.1 (10.6)	75.3 (10.8)	80.2 (10.8)	
Triglyceride, median (IQR)	79 (57–113)	118 (82–179)	86 (60–127)	131 (90–193)	
HDL-Cholesterol, median (IQR)	56 (48–65)	50 (43–58)	54 (46–63)	48 (41–56)	
HOMA-IR, median (IQR)	1.73 (1.38–2.18)	2.21 (1.74–2.82)	1.81 (1.45–2.28)	2.53 (1.94–3.40)	
Presence of Comorbidity^‡^, n(%)					
Yes	90 (1.9)	96 (4.2)	51 (1.7)	162 (3.3)	<0.001
No	4,501 (98.1)	2,215 (95.8)	2,905 (98.3)	4,787 (96.7)	

All values are presented as mean (SD) unless otherwise stated.

P value from ANOVA for continuous variables and Chi-Square test for categorical variables.

*MET minutes from IPAQ.

^†^Includes individuals who are students and housewives at the time of the health examination and survey.

^‡^Individuals with cancer, myocardial infarction, angina, hepatitis B or hepatitis C infection at the time of the health examination and survey.

Abbreviations: MET, Metabolic Equivalent Task; HOMA-IR, the Homeostasis Model Assessment of Insulin Resistance; SD, Standard Deviation; IQR, Interquartile Range; ANOVA, analysis of variance; IPAQ, International Physical Activity Questionnaire.

**Figure 1:**
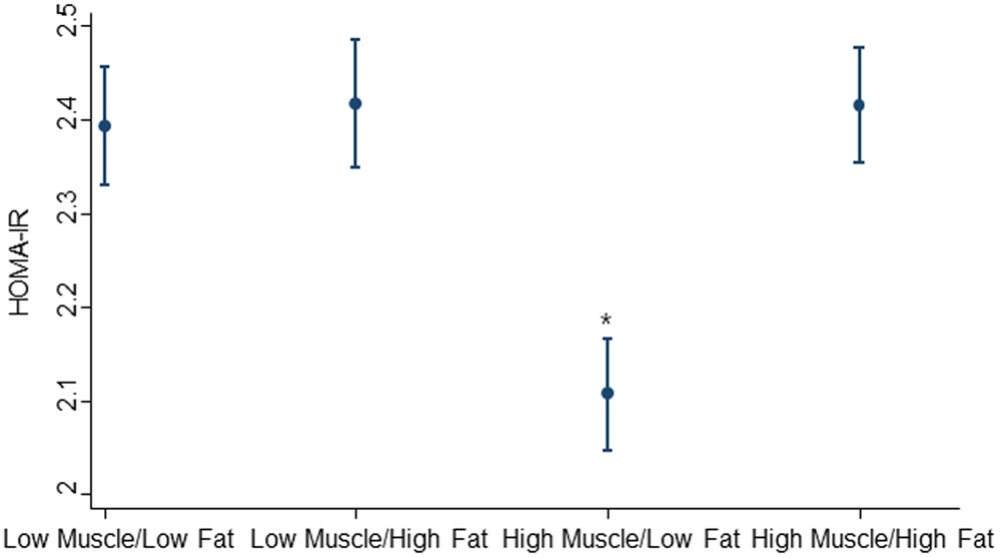
Least square mean of insulin resistance (HOMA-IR) by muscle mass and fat mass. Adjusted for sociodemographic factors (age, sex, education, employment status, area of residence, household income), health behavior (smoking, alcohol consumption, sleep duration, physical activity) and health-related status (BMI, and presence of comorbidity). Abbreviations: BMI, Body Mass Index; HOMA-IR, the Homeostasis Model Assessment of Insulin Resistance *P < 0.001 (reference group is low muscle/low fat).

Figure 1 displays least-square means of the Homeostasis Model Assessment of Insulin Resistance (HOMA-IR) among the participants by muscle mass and fat mass groups. High Muscle/low fat was associated with low insulin resistance (p < 0.001) when compared to low muscle/low fat after adjusting for sociodemographic, health behavior, and health-related status. No significant difference in insulin resistance was observed in the low muscle/high fat and high muscle/high fat group compared to low muscle/low fat group.

Table 2 demonstrates the association of muscle and fat mass with metabolic syndrome components. After adjustment for sociodemographic, health behavior, health-related, and dietary factors, we observed that participants in the low muscle/high fat (IRR:1.30; 95% CI:1.09–1.55, p < 0.01) and high muscle/high fat (IRR: 1.21; 95% CI:1.02–1.43, p < 0.05) categories had a higher incidence rate ratio for impaired fasting glucose (IFG) compared to low muscle/low fat participants. IRR for an elevated WC were significantly higher among participants belonging to the low muscle/high fat, high muscle/low fat, and high muscle/high fat groups when compared to the low muscle/low fat group. In those three groups, the high muscle/low fat group had the lowest IRR. There was no significant difference for high blood pressure in the abovementioned three types of body compositions. Participants in the low muscle/high fat category had a higher incidence of high TG (IRR:1.72; 95% CI:1.46–2.03, p < 0.001). This was also seen in the individuals in the high muscle/high fat category (IRR:1.55; 95% CI:1.30–1.84, p < 0.001). In addition, participants belonging to the low muscle/high fat (IRR:1.21; 95% CI:1.08–1.35, p < 0.01) and high muscle/high fat (IRR:1.17; 95% CI:1.05–1.30, p < 0.01) had a higher incidence for reduced HDL-cholesterol. The IRR for high TG and reduced HDL-cholesterol among participants in the high muscle/low fat group showed no statistical significance (IRR:1.05; 95% CI:0.89–1.24 for high TG and IRR:1.03; 95% CI:0.93–1.14 for reduced HDL-cholesterol, respectively) compared to the low muscle/low fat group after adjustments.

**Table 2:** Incidence rate ratio of (95% confidence intervals) for metabolic syndrome components by muscle mass and fat mass.

Components of Metabolic Syndrome*	Variable	Low Muscle/ Low Fat (*N* = *4,591*)	Low Muscle/ High Fat (*N* = *2,311*)	High Muscle/ Low Fat (*N* = *2,956*)	High Muscle/ High Fat (*N* = *4,949*)
Impaired Fasting Glucose	Proportion, n (%)	563 (12.3)	655 (28.3)	454 (15.4)	1,684 (34.0)
	Multivariable Model 1	1 (referent)	1.64 (1.41–1.93)***	1.18 (1.01–1.38)^*^	2.04 (1.80–2.30)***
	Multivariable Model 2	1 (referent)	1.26 (1.07–1.49)**	0.95 (0.81–1.11)	1.15 (0.98–1.35)
	Multivariable Model 3	1 (referent)	1.30 (1.09–1.55)**	0.97 (0.81–1.14)	1.21 (1.02–1.43)*
Elevated WC	Proportion, n (%)	81 (1.76)	459 (19.9)	124 (4.19)	2,743 (55.4)
	Multivariable Model 1	1 (referent)	10.5 (7.44–14.8)***	2.25 (1.52–3.34)***	30.3 (22.0–41.7)***
	Multivariable Model 2	1 (referent)	5.79 (4.06–8.24)***	1.66 (1.12–2.47)^*^	8.04 (5.60–11.6)^***^
	Multivariable Model 3	1 (referent)	6.13 (4.22–8.89)***	1.81 (1.20–2.75)**	8.76 (6.02–12.7)***
High Blood Pressure	Proportion, n (%)	322 (7.2)	389 (16.8)	319 (10.8)	1,031 (20.8)
	Multivariable Model 1	1 (referent)	1.35 (1.07–1.72)^*^	1.41 (1.14–1.74)**	2.26 (1.88–2.71)***
	Multivariable Model 2	1 (referent)	1.04 (0.81–1.33)	1.10 (0.88–1.37)	1.27 (0.99–1.63)
	Multivariable Model 3	1 (referent)	1.04 (0.80–1.36)	1.05 (0.83–1.33)	1.21 (0.94–1.58)
High Triglyceride	Proportion, n (%)	570 (12.4)	788 (34.1)	489 (16.5)	1,984 (40.1)
	Multivariable Model 1	1 (referent)	2.17 (1.88–2.50)***	1.16 (1.00–1.34)**	2.60 (2.31–2.93)***
	Multivariable Model 2	1 (referent)	1.66 (1.43–1.93)***	0.98 (0.84–1.14)	1.50 (1.28–1.77)***
	Multivariable Model 3	1 (referent)	1.72 (1.46–2.03)***	1.05 (0.89–1.24)	1.55 (1.30–1.84)***
Reduced HDL-cholesterol	Proportion, n (%)	1,543 (33.6)	1,119 (48.4)	1,042 (35.3)	2,721 (54.9)
	Multivariable Model 1	1 (referent)	1.48 (1.34–1.63)***	1.13 (1.03–1.24)^**^	1.68 (1.56–1.81)***
	Multivariable Model 2	1 (referent)	1.27 (1.14–1.41)***	1.04 (0.94–1.14)	1.20 (1.08–1.33)*
	Multivariable Model 3	1 (referent)	1.21 (1.08–1.35)**	1.03 (0.93–1.14)	1.17 (1.05–1.30)**

*Includes (1) Impaired Fasting Glucose (≥100 mg/dL), (2) Elevated WC (>90 cm for men and >85 cm for women), (3) High Blood Pressure (SBP: ≥130 mmHg and DBP: ≥85 mmHg), (4) High Triglyceride (≥150 mg/dL), (5) Reduced HDL-cholesterol (<50 mg/dL for men and <40 mg/dL for women).

Data presented are n (%) and IRR (95% CI).

Multivariable Model 1: Adjusted for age and sex.

Multivariable Model 2: Adjusted for age, sex, education, employment status, area of residence, household income, smoking, alcohol consumption, sleep duration, physical activity, BMI, and presence of comorbidity.

Multivariable Model 3: Adjusted for age, sex, education, employment status, area of residence, household income, smoking, alcohol consumption, sleep duration, physical activity, BMI, and presence of comorbidity, total energy intake, vitamin C, niacin, sodium, calcium, and fiber.

Abbreviations: IRR, Incidence Rate Ratio; CI, Confidence Interval WC, Waist Circumference; SBP, Systolic Blood Pressure; DBP, Diastolic Blood Pressure; HDL, High Density Lipoproteins; BMI, Body Mass Index.

Note: Each category of muscle mass and fat mass is compared to low muscle/low fat group (reference).

*p < 0.05, **p < 0.01, ***p < 0.001 (reference group is low muscle/low fat).

Table 3 shows the association of muscle mass and fat mass with the prevalence of metabolic syndrome. In the analysis conducted with our Model 3, participants in the low muscle/high fat category had a 1.9 times higher incidence rate ratio for metabolic syndrome (IRR = 1.90; 95% CI = 1.44–2.50) defined by NCEP ATP III criteria compared to those in the low muscle/low fat category. The incidence rate ratio for metabolic syndrome among participants who belonged to the high muscle/high fat category were 2.3 times higher in comparison to participants in the low muscle/low fat category. While the incidence rate ratio for metabolic syndrome was higher among the participants in low muscle/high fat and high muscle/ high fat groups, those in the high muscle/low fat category did not show a significantly higher incidence rate ratio (IRR 1.10; 95% CI = 0.82–1.47) when compared to those in the low muscle/low fat category. Table 4 lists the results of the sensitivity analysis. The overall results were consistent across the different categories of sociodemographic and health-related factors, but statistical significance was slightly attenuated in high education level (≥University/College), household income (highest third), former smoker, and short sleep duration (≤5 hours/day) groups.

**Table 3:** Incidence rate ratio of (95% confidence intervals) for metabolic syndrome based on the NCEP ATP III criteria by muscle mass and fat mass.

Variable	Low Muscle/ Low Fat (*N* = *4,591*)	Low Muscle/ High Fat (*N* = *2,311*)	High Muscle/ Low Fat (*N* = *2,956*)	High Muscle/ High Fat (*N* = *4,949*)
Proportion n (%)	159 (3.46)	462 (19.9)	188 (6.36)	1,780 (36.0)
Multivariable Model 1	1 (referent)	3.29 (2.56–4.22)***	1.45 (1.10–1.90)**	6.87 (5.53–8.53)***
Multivariable Model 2	1 (referent)	1.97 (1.51–2.56)***	1.07 (0.81–1.41)	2.29 (1.77–2.96)***
Multivariable Model 3	1 (referent)	1.90 (1.44–2.50)***	1.10 (0.82–1.47)	2.30 (1.76–3.00)***

Data presented are n (%) and IRR (95% CI).

Criteria for metabolic syndrome was defined as meeting three or more of the following conditions as suggested by the NCEP ATP III: (1) Impaired Fasting Glucose (≥100 mg/dL), (2) Elevated WC (>90 cm for men and >85 cm for women), (3) High Blood Pressure (SBP: ≥130 mmHg and DBP: ≥85 mmHg), (4) High Triglyceride (≥150 mg/dL), (5) Reduced HDL-cholesterol (<50 mg/dL for men and <40 mg/dL for women).

Multivariable Model 1: Adjusted for age and sex.

Multivariable Model 2: Adjusted for age, sex, education, employment status, area of residence, household income, smoking, alcohol consumption, sleep duration, physical activity, BMI, and presence of comorbidity.

Multivariable Model 3: Adjusted for age, sex, education, employment status, area of residence, household income, smoking, alcohol consumption, sleep duration, physical activity, BMI, and presence of comorbidity, total energy intake, vitamin C, niacin, sodium, calcium, and fiber.

Abbreviations: IRR, Incidence Rate Ratio; CI, Confidence Interval; WC, Waist Circumference; SBP, Systolic Blood Pressure; DBP, Diastolic Blood Pressure; HDL, High Density Lipoproteins;

NCEP ATP III, National Cholesterol Education Program-Adult Treatment Panel III;

Note: Each category of muscle mass and fat mass is compared to low muscle/low fat group (reference).

*p < 0.05, **p < 0.01, ***p < 0.001 (reference group is low muscle/low fat).

**Table 4:** Subgroup Analysis for the prevalence of metabolic syndrome based on ATP III criteria by muscle mass and fat mass.

	Low Muscle/Low Fat	Low Muscle/High Fat	High Muscle/Low Fat	High Muscle/High Fat
Sex				
Male	1 (referent)	1.65 (1.15-2.38^**^	0.94 (0.65-1.35)	1.73 (1.19–2.50)**
Female	1 (referent)	2.13 (1.41–3.21)***	1.30 (0.82–2.05)	3.00 (2.04–4.40)***
Age Group (years)				
18–43	1 (referent)	2.22 (1.27–3.92)**	0.76 (0.42–1.36)	3.46 (2.13–5.61)***
44–65	1 (referent)	1.95 (1.43–2.68)***	1.34 (0.95–1.87)	2.08 (1.50–2.87)***
Education Level				
≤Elementary	1 (referent)	2.32 (1.38–3.91)**	1.35 (0.75–2.43)	2.58 (1.55–4.31)***
Middle/High School	1 (referent)	1.94 (1.35–2.78)***	1.15 (0.79–1.68)	2.37 (1.69–3.33)***
≥University/College	1 (referent)	1.25 (0.65–2.41)	0.81 (0.43–1.53)	1.84 (0.96–3.51)
Employment Status				
Employed	1 (referent)	1.70 (1.20–2.40)**	1.04 (0.73–1.49)	1.97 (1.40–2.76)***
Unemployed	1 (referent)	2.04 (1.31–3.18)**	1.13 (0.69–1.87)	2.59 (1.72–3.91)***
Residential Area				
Capital	1 (referent)	1.86 (1.10–3.17)*	1.17 (0.67–2.05)	2.14 (1.26–3.62)**
Metropolitan	1 (referent)	2.36 (1.17–4.78)*	1.67 (0.80–3.48)	2.97 (1.49–5.89)**
Town/City	1 (referent)	1.77 (1.22–2.57)**	0.91 (0.61–1.34)	2.24 (1.55–3.22)***
Household Income				
Lowest Third	1 (referent)	3.05 (1.61–5.76)**	1.99 (1.00–3.98)*	2.75 (1.41–5.35)**
Middle Third	1 (referent)	1.98 (1.35–2.91)***	1.19 (0.80–1.75)	2.55 (1.79–3.63)***
Highest Third	1 (referent)	1.25 (0.78–2.00)	0.65 (0.39–1.08)	1.50 (0.95–2.36)
Smoking				
Non–Smoker	1 (referent)	2.00 (1.32–3.04)**	1.22 (0.78–1.90)	2.94 (1.96–4.41)***
Former Smoker	1 (referent)	1.38 (0.86–2.21)	0.76 (0.47–1.24)	1.43 (0.91–2.26)
Current Smoker	1 (referent)	2.85 (1.50–5.42)**	1.61 (0.82–3.14)	2.80 (1.48–5.29)**
Alcohol Consumption				
Drinker	1 (referent)	2.37 (1.51–3.61)***	1.60 (1.00–2.57)	3.30 (2.18–5.00)***
Non–Drinker	1 (referent)	1.67 (1.18–2.37)**	0.87 (0.61–1.24)	1.77 (1.26–2.49)**
Sleep Duration (hours)				
≤5	1 (referent)	1.54 (0.66–3.58)	1.24 (0.53–2.90)	2.27 (1.03–5.01)*
6	1 (referent)	2.18 (1.17–4.05)*	0.94 (0.49–1.79)	2.19 (1.16–4.12)*
7	1 (referent)	1.49 (0.95–2.37)	0.85 (0.52–1.40)	1.72 (1.11–2.68)*
8	1 (referent)	2.13 (1.20–3.76)**	1.65 (0.93–2.91)	3.01 (1.76–5.16)***
≥9	1 (referent)	2.20 (1.00–4.83)*	0.86 (0.64–2.18)	2.40 (1.23–4.67)*
Physical Activity				
(MET-min)				
Low (≤600)	1 (referent)	2.65 (1.46–4.79)**	1.45 (0.77–2.71)	3.63 (2.02–6.54)***
Moderate (≥601–2,999)	1 (referent)	1.53 (1.00–2.34)*	0.82 (0.51–1.31)	1.77 (1.15–2.73)**
High (≥3,000)	1 (referent)	1.96 (1.22–3.15)**	1.31 (0.82–2.10)	2.34 (1.47–3.74)***

Data presented are IRR (95% CI).

All IRR (95% CI) presented above are from multivariable model 3 (adjusted for for age, sex, education, employment status, area of residence, household income, smoking, alcohol consumption, sleep duration, physical activity, BMI, and presence of comorbidity, total energy intake, vitamin C, niacin, sodium, calcium, and fiber).

Criteria for metabolic syndrome was defined as meeting three or more of the following conditions as suggested by the NCEP ATP III: (1) Impaired Fasting Glucose (≥100 mg/dL), (2) Elevated WC (>90 cm for men and >85 cm for women), (3) High Blood Pressure (SBP: ≥130 mmHg and DBP: ≥85 mmHg), (4) High Triglyceride (≥150 mg/dL), (5) Reduced HDL-cholesterol (<50 mg/dL for men and <40 mg/dL for women).

*Categorized as normal (<22.9 kg/m^2^), overweight (23–24.9 kg/m^2^), obese (25–29.9 kg/m^2^), severely obese (≥30 kg/m^2^) based on WHO criteria for Asian population.

Abbreviations: BMI, Body Mass Index; WHO, World Health Organization; IRR, Incidence Rate Ratio; CI, Confidence Interval; WC, Waist Circumference; SBP, Systolic Blood Pressure; DBP, Diastolic Blood Pressure; HDL, High Density Lipoproteins; NCEP ATP III, National Cholesterol Education Program-Adult Treatment Panel III;

Note: Each category of muscle mass and fat mass is compared to low muscle/low fat group (reference).

*p < 0.05, **p < 0.01, ***p < 0.001 (reference group is low muscle/low fat).

## Discussion

Based on the nationally representative cross-sectional study of Korean adults, low muscle/high fat and high muscle/high fat were associated with IFG, elevated WC, high TG, reduced HDL-cholesterol, and also with the prevalence of metabolic syndrome as compared with low muscle/low fat after adjusting for sociodemographic and health-related factors. Also, insulin resistance adjusted for potential confounders was significantly lower in high muscle/low fat group compared to low muscle/low fat group. The findings of this study suggest that accumulation of abdominal fat is associated with insulin resistance and metabolic syndrome regardless of muscle mass.

Previous epidemiological studies have addressed the issue of fat mass and muscle mass in relation to insulin resistance and metabolic syndrome in both Asian and Western population. In a study by Srikanthan *et al*.^12^ based on the third NHANES, the participants in the highest quartile of skeletal muscle had a reduction of HOMA-IR compared to the lowest quartile of skeletal muscle. A cross-sectional study of Korean men and non-pregnant women found that SAT is inversely related to the incidence of metabolic syndrome after adjusting for VAT based on the data from patients free of chronic diseases^9^. The difference in the level of cytokine secretion between SAT and VAT adipose tissue support this association^13,14^. Additionally, Burrows *et al*. found in a Chilean birth cohort that low muscle mass is associated with cardiometabolic risk factors independent of dietary intake^15^.

While findings in our study are similar to the previous reports, it should be noted that our study included Korean adults aged between 18 and 65 years. We found that the overall association remained consistent in the participants of different age groups. This might be explained in part from excluding the elderly (age >65 years) participants from our study. Recent epidemiological studies have shown that the odds of metabolic syndrome was 6 to 8 times higher in postmenopausal Korean women, elderly Korean men and women, and adult Caucasian subjects with sarcopenic obesity (decline of muscle mass and increase of fat mass with aging) compared to those without sarcopenic obesity^16–19^. Therefore, including the elderly population (>65 years of age) in our analysis might have caused overestimation on the association of low muscle/high fat with metabolic syndrome due to the possible confounding effect from sarcopenic obesity.

Few mechanisms may explain the combined effect on muscle mass and fat mass on insulin resistance and metabolic syndrome. Since insulin-induced glucose uptake occurs in skeletal muscle, high muscle mass might result in a stable control over glucose levels^20^. The protective effect of an increase in muscle mass on insulin resistance and metabolic syndrome has been shown in individuals with and without diabetes^21,22^. On the other hand, it has been suggested that excessive and naturally occurring deposition of adipose tissue in the abdomen may increase the risk of metabolic syndrome^23^. Because the regulatory function of energy storage in subcutaneous adipose tissue is limited, excessive chemical energy flow to liver and skeletal muscles can cause metabolic disturbances^24^. In addition, a study conducted among non-diabetic men in Finland showed that an increased accumulation of hepatic fat is linked to an abnormal amount of lipids in the blood, and hepatic insulin resistance^25^. Increased levels of intramyocellular lipids have been shown to elevate the insulin resistance of skeletal muscles^26^. Thus, protective association of muscle mass with metabolic syndrome may have been attenuated in high muscle/high fat group.

This study has some limitations that need to be addressed. First, the results of our findings cannot be regarded as a direct cause-effect relationship between proportion of body composition, insulin resistance, and metabolic syndrome due to the cross-sectional design of this study. Second, there were group differences in our analytic sample (e.g. twice as many women in the reference group and socioeconomic differences) that were only adjusted and stratified in the analyses.

Third, there were additional factors associated with metabolic syndrome that were not provided in our dataset (e.g. health-related quality of life such as parameters of mental health). Therefore, in this study, we could not account for the effect from other confounding factors that were unavailable in the KNAHNES dataset. Despite the limitations from the study design and data, the strength of our study was the assessment of the association between the proportion of DEXA scan-measured muscle and fat mass to insulin resistance and the prevalence of metabolic syndrome in a nationally representative sample.

## Conclusion

In summary, the results of our study suggest the clinical significance of muscle mass and fat mass with insulin resistance and metabolic syndrome in Korean adults. Whether this concept is widely applicable to other ethnic groups or not needs further investigation due to the ethnic heterogeneity in body composition and health outcomes^27^. Well-designed cohort studies or randomized control studies are necessary to investigate the combined influence of muscle mass and fat mass to validate the findings of this study.

## Methods

### Ethics Statement

All the participants provided written informed consent before the KNHANES IV-V began. The Institutional Review Board at the Seoul National University Hospital, which is in accordance with the Declaration of Helsinki, approved this study.

### Study Participants

We used the KNHANES IV-V to assess the cross-sectional relationship between different body composition types, insulin resistance, and metabolic syndrome. KNHANES IV-V data were collected using a multistage, probability-cluster survey method for the sample to represent the entire population of the Republic of Korea from 2008 to 2011. The KNHANES was conducted by the Korea Center for Disease Control and Prevention (KCDC) under the guidance of the Ministry of Health and Welfare. The validity of this nationally representative data has been described in previous studies^28,29^. The inclusion criteria for this cross-sectional study was 15,378 participants of the KNHANES aged between 18 years and 65 years who received a whole-body DEXA scan. We excluded the participants who reported that they took medications (antihypertensive, antihyperglycemic, and antihyperlipidemic agents) (N = 98) and had less than 8 hours of fasting time (N = 473) prior to the health examination from the study sample. Therefore, 14,807 participants who met the inclusion criteria were selected for this study.

### Assessment of Fat Mass and Muscle Mass

Based on the whole-body DEXA scan dataset of KNHANES (measured and recorded by credible health professionals), we derived trunk fat mass index (TFMI) and appendicular muscle (lean mass of arms and legs combined) mass index (AMMI) for categorization. We calculated TFMI from trunk fat mass in kilograms (kg) divided by height in meters squared (m^2^). Similarly, we calculated AMMI using appendicular muscle mass (kg) and height (m^2^). We chose TFMI because trunk fat mass represents the level of visceral adipose tissue, which is a more accurate predictor of metabolic risk^30^ compared to the whole-body fat mass index (FMI). We created four different gender-specific body composition groups based on the following: (1) Low Muscle/Low Fat: AMMI < median and TFMI < median, (2) Low Muscle/High Fat: AMMI < median and TFMI ≥ median, (3) High Muscle/Low Fat: AMMI ≥ median and TFMI < median, and (4) High Muscle/High Fat: AMMI ≥ median and TFMI ≥ median. In addition, we linked the health survey data containing sociodemographic status and health behavior and the laboratory measurement data to the DEXA measurement dataset. The details and validity of creating the body composition types to study the combined effect of muscle mass and fat mass on health outcomes such as cardiovascular disease and mortality have been previously described^31,32^.

### Assessment of Insulin Resistance and Metabolic Syndrome

Data from health examination and laboratory test were used to assess insulin resistance and metabolic syndrome across the participants of different body composition types. Insulin resistance index was assessed through HOMA-IR by employing the level of fasting insulin and glucose^33,34^. We adopted the criteria for metabolic syndrome from the NCEP/ATP III, which was adjusted for Asian-Pacific populations including Koreans^35^. Criteria for metabolic syndrome was defined as meeting three or more of the following conditions: (1) impaired fasting glucose (≥100 mg/dL), (2) elevated waist circumference (≥90 cm for men and ≥85 cm for women), (3) high blood pressure (SBP: ≥130 mmHg and DBP: ≥85 mmHg), (4) high triglyceride (≥150 mg/dL), (5) reduced HDL-cholesterol (<50 mg/dL for men and <40 mg/dL for women).

### Assessment of Confounding Factors

We used survey data of the KNHANES to assess confounding factors in this study. Information on sociodemographic factors (age, sex, education level employment status, residential area, household income) and health-related factors (smoking status, alcohol consumption, physical activity, nutritional intake, and presence of comorbidity) was collected from self-reported questionnaires.

### Statistical Analyses

We examined the sociodemographic and clinical characteristics of the study population across different categories of body compositions grouped into low muscle/low fat, low muscle/high fat, high muscle/low fat, and high muscle/high fat. Analysis of variance (ANOVA) and the chi-square test was used for the comparison of general characteristics across different categories of body composition type for continuous and categorical variables, respectively. To account for pre-diagnosed medical conditions with significant impact on metabolic syndrome (cancer, myocardial infarction, angina, hepatitis B, and hepatitis C), we grouped the participants into the following categories: (1) those who reported that they have been diagnosed with any of the above mentioned conditions and currently receiving treatment (2) those without comorbidity associated with metabolic syndrome. As a part of multivariate analysis, we adjusted for the presence of a comorbidity known to be related to metabolic syndrome as a categorical variable. To assess the relationship between body composition types and insulin resistance (HOMA-IR), we estimated the least-square mean (marginal means) according to types of body composition. Using negative binomial regression, we computed an incidence rate ratio and 95% confidence interval (IRR; 95% CI) for the prevalence of metabolic syndrome components and metabolic syndrome defined by NCEP-ATPIII criteria. In the multivariate models, we constructed a minimally adjusted model (adjusted for age and sex) as Model 1. In Model 2, we built a model that was adjusted for sociodemographic, health behavior, and health-related factors (age, sex, level of education, employment status, area of residence, household income, smoking, alcohol consumption, sleep duration, physical activity, BMI, and the presence of comorbidities). In addition, we built a Model 3, which included dietary factors (total energy intake, vitamin C, niacin, sodium, calcium, and fiber consumption). We performed a sensitivity analysis by calculating IRR (95% CI) across different categories of relevant factors that were used to build different models in this analysis. We collected the KNHANES data using SAS, version 9.4 (SAS Institute Inc., Cary, NC, USA) and performed statistical analyses using STATA, version 14 (STATA Corp., College Station, TX, USA). We used survey data commands “svy” in STATA to incorporate the complex sampling weights into the results. Therefore, the results were weighted to represent the entire non-institutionalized civilian population of the Republic of Korea. The level of statistical significance for all results was set to p < 0.05.

### Data availability statement

No additional data available.
